# The harmful effect of ankylosing spondylitis on diabetes mellitus: new evidence from the Mendelian randomization analysis

**DOI:** 10.3389/fendo.2024.1369466

**Published:** 2024-11-22

**Authors:** Zheng Ren, Liang He, Jing Wang, Li Shu, Chenyang Li, Yuan Ma

**Affiliations:** ^1^ Xinjiang Institute of Spinal Surgery, Sixth Affiliated Hospital of Xinjiang Medical University, Urumqi, China; ^2^ Institute of General Surgery, Wulumuqi General Hospital of People’s Liberation Army (PLA), Urumqi, China; ^3^ Micro Operation of the Third People’s Hospital of Xinjiang Uygur Autonomous Region, Urumqi, China

**Keywords:** ankylosing spondylitis, type 1 and 2 diabetes mellitus, glucose metabolism, Mendelian randomization, meta-analysis

## Abstract

**Background:**

While observational research has highlighted a possible link between ankylosing spondylitis (AS) and type 2 diabetes (T2DM), the quality of evidence remains limited, and the causal relationship is yet to be established. This study aims to explore the causal link between AS and T2DM, as well as its impact on traits related to glucose metabolism.

**Method:**

To infer a causal relationship between AS and various diabetes-related traits, including type 1 diabetes (T1DM), T2DM, blood glucose levels, fasting glucose, glycated hemoglobin, and fasting insulin, we employed Mendelian randomization (MR) analysis. We sourced GWAS summary data for both exposure and outcome variables from the IEU OpenGWAS database, GWAS Catalog, and FinnGen database. To synthesize the results of the MR analyses, we applied meta-analysis techniques using either a fixed or random effects model. For identifying and excluding instrumental variants (IVs) that exhibit horizontal pleiotropy with the outcomes, we utilized the MR-PRESSO method. Sensitivity analyses were conducted using the MR-Egger method, along with Q and I^2 tests, to ensure the robustness of our findings.

**Results:**

Our analysis revealed a significant association between AS and an increased risk of T1DM with an odds ratio (OR) of 1.5754 (95% CI: 1.2935 to 1.9187) and T2DM with an OR of 1.0519 (95% CI: 1.0059 to 1.1001). Additionally, AS was associated with elevated levels of fasting glucose (beta coefficient = 0.0165, 95% CI: 0.0029 to 0.0301) and blood glucose (beta coefficient = 0.0280, 95% CI: 0.0086 to 0.0474), alongside a decrease in fasting insulin levels (beta coefficient = -0.0190, 95% CI: -0.0330 to -0.0050).

**Conclusion:**

Our findings collectively underscore the detrimental impact of AS on the development of diabetes, highlighting the critical influence of autoimmune disorders in diabetes onset. This provides profound insights into the pathogenesis of diabetes from an immunological standpoint.

## Introduction

Ankylosing spondylitis (AS) is classified as an autoimmune condition characterized by chronic inflammation and is often considered a type of spondyloarthritis (SpA) (1). Typically, individuals with AS experience progressive back pain and impairment of axial joints, notably the hips, though peripheral joints may also be affected (2). Beyond joint issues, AS patients frequently contend with extra-articular symptoms such as uveitis, psoriasis, and inflammatory bowel disease (IBD), which accompany the disease’s progression (3). Epidemiological data indicate that AS predominantly emerges around the age of 30, with notable gender disparities in incidence rates and symptomatology (4). For instance, females with AS tend to encounter more severe active phases and fatigue more rapidly compared to males (5). Despite advances in early detection and diagnosis, treating AS remains a considerable challenge due to the complex and not fully understood pathophysiology of the disease (6). The development of AS is influenced by a multifaceted interplay of genetic, environmental, and immunological factors (7). Disruptions in both the innate and adaptive immune systems, triggered by genetic and environmental factors, are crucial in AS’s pathology (8). Growing evidence indicates that the immune dysregulation seen in patients with ankylosing spondylitis (AS) not only impacts joint health but also disrupts the balance of other bodily systems, thereby increasing the risk of various conditions, including inflammatory bowel disease (IBD), myelodysplastic syndrome, and cardiovascular mortality (9–11). Furthermore, the alterations in glucose metabolism associated with ankylosing spondylitis underscore the complexity of this condition beyond its inflammatory symptoms. Understanding these metabolic changes is essential for developing targeted therapies and enhancing patient management strategies (12).

As of now, over 400 million individuals worldwide have been diagnosed with diabetes, and projections suggest this number could rise to 642 million by 2040, representing a significant and growing global public health challenge (13). Diabetes, a chronic condition marked by disturbances in energy metabolism, can be broadly categorized into type 1 diabetes mellitus (T1DM), type 2 diabetes mellitus (T2DM), and other subtypes based on the underlying mechanisms involved (13). T1DM primarily results from autoimmune-mediated damage to the pancreas (14), whereas type 2 diabetes mellitus (T2DM) is often linked to metabolic disturbances related to obesity and unhealthy dietary habits (15). Recent research has highlighted the significant impact of immune system dysregulation in the development of T2DM (16). For instance, studies have indicated that chronic activation of IL-1, a key player in the innate immune response, can adversely affect the development of type 2 diabetes. This is due to elevated levels of IL-1β, which are known to provoke inflammation and impair the function of β-cells (17). Similarly, Biondi and colleagues have documented that thyroid malfunctions stemming from immune disturbances are linked to a heightened risk of diabetes (18). These research findings enhance our comprehension of diabetes’ pathogenesis through the lens of immune-related conditions, indicating a possible connection between autoimmune diseases and diabetes.

Therefore, the detrimental impact of ankylosing spondylitis (AS) on various bodily systems has sparked our curiosity in examining if AS influences diabetes or glucose metabolism in any way. Notably, two observational studies have highlighted a positive correlation between AS and T2DM (19, 20). Nonetheless, the evidence provided by these observational studies is of a low level, and the presence of unforeseen confounding factors could distort their results. Moreover, determining a causal relationship between AS and diabetes based solely on observational data is challenging. Recently, Mendelian randomization (MR) analysis has emerged as a powerful method for exploring the causal links between exposures and outcomes, offering a more reliable approach to understanding these associations (21). In MR analysis, instrumental variables (IVs), which consist of a collection of single nucleotide polymorphisms (SNPs), are used to mimic the exposure condition. This approach is analogous to randomly allocating individuals to either a treatment or control group, as is done in randomized controlled trials (RCTs) (22). Moreover, MR has the advantage over traditional observational epidemiological studies in that it can significantly reduce the impact of confounding factors, thereby diminishing the biases typically associated with observational research (23). MR serves as a valuable tool for understanding the complex interplay between autoimmune diseases and various conditions, including metabolic disorders like sarcopenia (24, 25), bronchiectasis (26), and COVID-19 (27). Additionally, variants in TLR genes have been linked to several autoimmune conditions, including type 1 diabetes (T1D), Graves’ disease, rheumatoid arthritis, systemic lupus erythematosus (SLE), and multiple sclerosis. These SNPs can disrupt essential signaling pathways, contributing to increased susceptibility to autoimmune disorders (28). The insights gained from these studies underscore the importance of genetic factors in determining disease risk and may inform future research directions aimed at developing targeted interventions for individuals with autoimmune disorders. In our research, we utilized a two-sample MR approach to examine the causal link between AS and diabetes. To our knowledge, this is the inaugural study to explore the causal relationship between AS and diabetes using MR analysis, potentially offering crucial insights into the mechanisms underlying diabetes.

## Data and methods

### Study design

**Figure 1:**
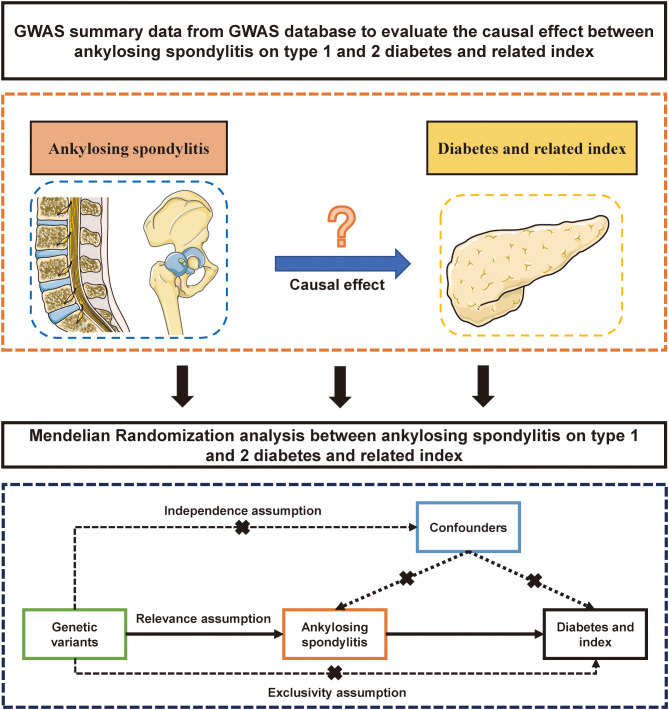
The flow gram of mendelian randomization analysis between ankylosing spondylitis on diabetes mellitus.

This research aims to uncover the causal relationship between AS and diabetes, including its related traits, through a two-sample MR analysis. Compared to traditional clinical studies, MR utilizes instrumental variables to assess causal relationships between two factors. For topics where conducting clinical research is particularly challenging, MR studies offer a unique advantage as pioneering exploratory tools. Thus, we considered AS as the exposure and outcomes such as T1DM and T2DM, fasting glucose, fasting insulin, blood glucose, and glycated hemoglobin. Genetic instrumental variables (IVs) were selected to represent AS exposure. In cases where multiple datasets were analyzed using MR, a meta-analysis was conducted to aggregate the results of the MR studies. A detailed schematic flowchart outlining the study’s methodology is presented in **Figure 1**.

### Source of GWAS summary dataset

For this study, we utilized the most extensive genome-wide association study (GWAS) summary data available for both the exposure and outcomes, drawing from previous GWAS research. **Table 1** illustrates that the exposure GWAS summary data focused on AS, with an IEU GWAS ID of ebi-a-GCST005529 (Ncase=9069) (29). The outcome GWAS summary datasets covered diabetes and its related traits, sourced from the IEU GWAS database (https://gwas.mrcieu.ac.uk/), the FinnGen database (https://www.finngen.fi/en/access_results), and the UK Biobank database (http://www.nealelab.is/faq). These outcome datasets include T1DM, T2DM, blood glucose, fasting glucose, fasting insulin, and glycated hemoglobin, with sample sizes ranging from six thousand to three hundred thousand. To minimize the potential for ethnic stratification bias, our analysis was restricted to the European population. Further details and the summary data used in our study are provided in **Table 1**.

**Table 1:** The datasets used in this study from IEU GWAS database and GWAS Catalog database.

Explore or outcomes	PMID	IEUID or GWASID	Ncase	Ncontrol
Explore				
Ankylosing spondylitis	23749187	ebi-a-GCST005529	9,069	13578
Outcomes				
T2D	30054458	ebi-a-GCST006867	61,714	593952
	32499647	ebi-a-GCST010118	77,418	356,122
	29632382	ebi-a-GCST007515	48,286	250,671
	29358691	ebi-a-GCST005413	12,931	57,196
	22885922	ieu-a-24	34,840	114,981
	24509480	ieu-a-23	26,488	83,964
		FinnGen_R9_T2D	57698	308252
T1D	25751624	ebi-a-GCST005536	6,683	12,173
	32005708	ebi-a-GCST010681	9,266	15,574
	33830302	ebi-a-GCST90000529	7,467	10,218
		finn-b-T1D_WIDE	6,729	182,573
Blood glucose	22581228	ebi-a-GCST005186	58,074	
	20081858	ebi-a-GCST000568	46,186	
	25625282	ebi-a-GCST007858	33,231	
Fasting glucose	34059833	ebi-a-GCST90002232	200,622	
	22885924	ieu-b-114	133,010	
	22581228	ieu-b-113	58,074	
Fasting insulin	34059833	ebi-a-GCST90002238	151,013	
	22885924	ieu-b-116	108,557	
	22581228	ieu-b-115	51,750	
	20081858	ebi-a-GCST000571	38,238	
	25625282	ebi-a-GCST007857	30,825	
	31217584	ebi-a-GCST008033	12,687	
Glycated hemoglobin	34059833	ebi-a-GCST90002244	146,806	
		ukb-d-30750_irnt	344182	

T1D, Type 1 diabetes; T2D, Type 2 diabetes.

### SNP selection and two-sample Mendelian randomization analysis

We employed the inverse variance weighted (IVW) method, augmented with multiplicative random effects, to assess the causal influence of AS on diabetes and related metabolic traits. Single nucleotide polymorphisms (SNPs) from GWAS summary data served as the genetic instruments for MR analysis. It is crucial that three key assumptions are met before proceeding with MR analysis: 1) The genetic instruments must be significantly associated with the exposure, meaning the SNPs chosen for AS must have a p-value less than 5e-8, indicating a strong association with the exposure. 2) The selected SNPs should not have a strong association with the outcomes (diabetes and diabetes-related traits) independent of the exposure, with p-values for these outcomes being greater than 5e-5. 3) The genetic instruments must influence the outcomes solely through their effect on the exposure, ensuring that the identified SNPs impact diabetes and its related traits only via AS. To adhere to these principles, we implemented several strategies for selecting SNPs: 1) We applied an LD clustering algorithm (30) with parameters set to P ≤ 5e-8 and r2 = 0.001 to exclude SNPs that may introduce bias due to linkage disequilibrium (LD), thereby minimizing complex LD effects. 2) We restricted the gene windows of SNPs to 100 kb to enhance the precision of our analysis. 3) To mitigate the risk of pleiotropy, which could skew results, we excluded SNPs if five or more were related to the exposure. 4) We calculated the F-value to evaluate the strength of the association between the IVs and both AS and the diabetes-related traits. A standard F-value of more than 20 was used to identify robustly associated SNPs, ensuring the efficacy of the genetic instruments. These meticulous screening methods for SNPs ensure the integrity and reliability of our MR analysis, following the formula provided in reference (31).


(1) $$\text{F}={\text{R}}^{2}\left(\text{n}-1-\text{k}\right)/\left(1-{\text{R}}^{2}\right)\text{k}$$

Where R2 is represented as instrumental variance, the variate n represents the sample size. The k represented the number of IVs.

When only a single SNP was identified, the Wald ratio method was employed to estimate the causal effect of this specific situation. For instances where the number of SNPs fell between 1 and 3, we implemented a fixed effect model to analyze the data. Conversely, when the number of selected SNPs was 3 or more, we adopted a random effects model alongside the IVW method for MR analysis. The odds ratio (OR) and 95% confidence intervals (CI) were calculated to assess the strength and precision of the causal relationship, with a p-value of less than 0.05 indicating statistical significance. For analyses involving four or more SNPs, the MR-PRESSO test was applied to detect and correct for horizontal pleiotropy, which occurs when genetic variants influence multiple traits in a manner not mediated through the trait of interest. Additionally, to ensure the robustness and reliability of our findings, sensitivity analyses were conducted. These analyses help confirm that the observed associations are not driven by pleiotropic effects of the selected genetic instruments and that the MR results are consistent under different statistical models.

### Meta-analysis

To obtain accurate results, we used two types of meta-analysis models in our study: fixed-effect and random-effect models. The choice of model depended on the similarity of the datasets. We used the fixed-effect model when the studies were similar, assuming a consistent effect size across all studies. Conversely, we used the random-effect model when there was significant variability among the studies, accounting for differences both within and between studies. This approach ensures that our conclusions are robust and consider variations in study populations and designs. Our meta-analysis followed the PRISMA guideline (32). Related studies used specific search terms for retrieval from PubMed, Embase, Cochrane Library: (“spondylitis, ankylosing”[MeSH Terms] OR (“spondylitis”[All Fields] AND “ankylosing”[All Fields]) OR “ankylosing spondylitis”[All Fields] OR (“ankylosing”[All Fields] AND “spondylitis”[All Fields])) AND (“diabetes mellitus”[MeSH Terms] OR (“diabetes”[All Fields] AND “mellitus”[All Fields]) OR “diabetes mellitus”[All Fields]).

### Sensitivity analysis

To ensure the robustness and validity of the MR analysis investigating the impact of AS on diabetes and its related metabolic traits, we employed a comprehensive suite of statistical tests, including MR-PRESSO, Egger-intercept test, Cochrane’s Q-test, heterogeneity I2, and the leave-one-out sensitivity analysis. The MR-PRESSO test was specifically utilized to identify and adjust for any horizontal pleiotropy within our MR findings, initiating a global test for pleiotropy whenever the p-value fell below 0.05. Subsequent to this analysis, SNPs implicated in horizontal pleiotropy were excluded unless their removal resulted in the absence of SNPs for analysis. The Egger-intercept test was then applied to further probe for pleiotropic effects within the MR framework, with a p-value greater than 0.05 indicating an absence of pleiotropic bias. Additionally, Cochrane’s Q-test and the calculation of heterogeneity I2 were conducted to evaluate the variance in effect sizes across different studies, highlighting significant heterogeneity with p-values less than 0.05 and quantifying it in percentage terms through I2. Finally, the leave-one-out sensitivity analysis was performed to test the influence of individual SNPs on the overall MR results, ensuring the stability and reliability of the causal inference by sequentially removing each SNP and recalculating the MR estimates. These analyses were conducted using the R statistical software, supplemented by various packages tailored for Mendelian randomization studies, thereby providing a thorough and reliable assessment of the causal relationship between AS and diabetes-related outcomes.

### Ethical statement

The GWAS summary data utilized in this research were obtained from a public database, originally sourced from published studies. These studies had previously received ethical approval from their respective institutional ethics committees, including the completion of informed consent procedures. Consequently, our study did not require further ethical clearance.

## Results

### Effect of AS on T1DM

**Figure 2:**
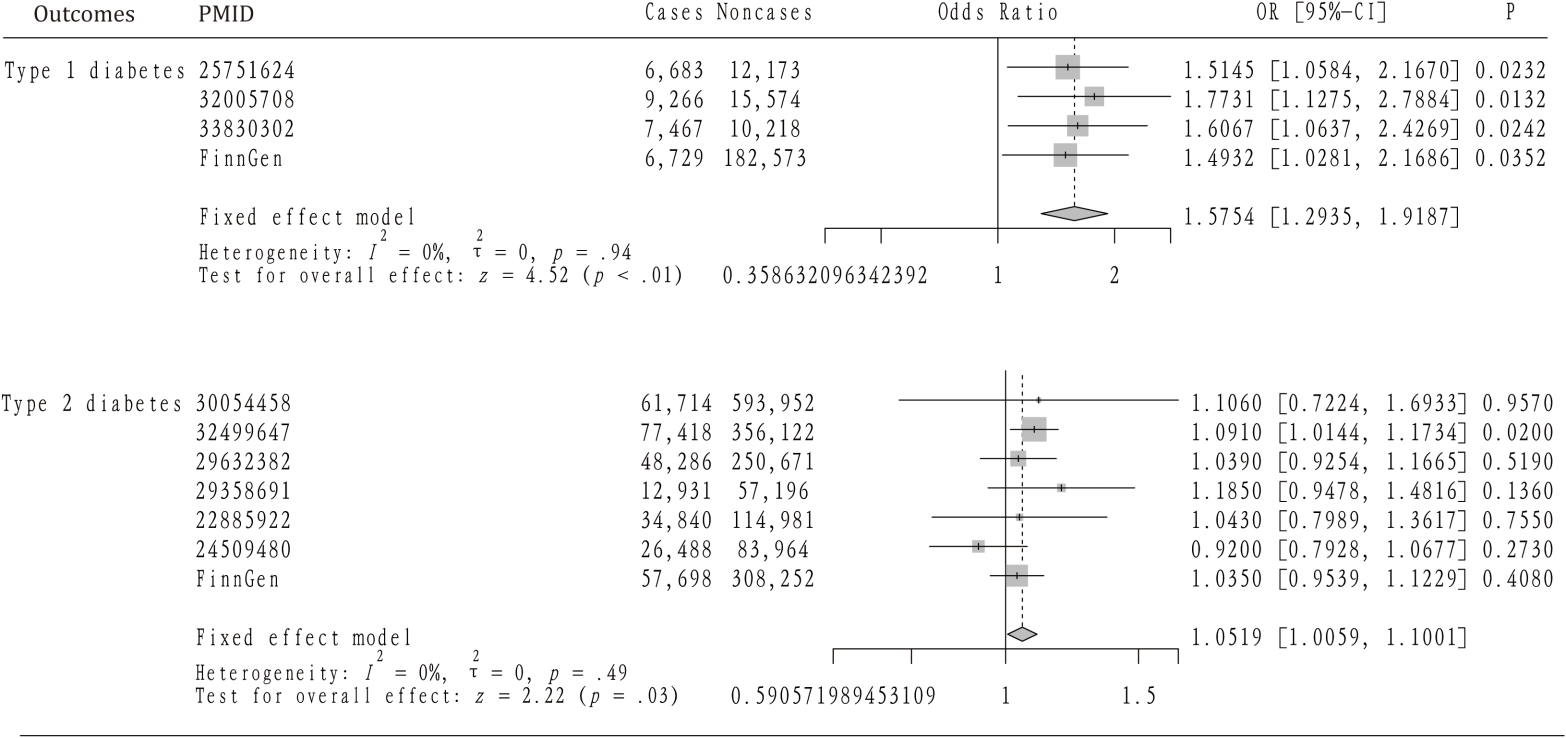
The mendelian randomization analysis and meta-analysis about the casual effect of ankylosing spondylitis on type 1 and 2 diabetes mellitus.

**Table 1** provides a summary of the baseline GWAS data on type 1 diabetes, including four datasets that encompass 30,175 individuals with AS and 220,538 controls. **Figure 2** illustrates the results from the MR analysis conducted using the IVW method, which revealed a consistently positive association between AS and the risk of T1DM across all datasets. Given the lack of significant heterogeneity among the results from the four MR analyses (I2 = 0%, p=0.94), a fixed-effect model was applied to aggregate the findings. The pooled results from the meta-analysis demonstrated that AS significantly elevates the risk of developing T1DM (OR=1.5754, 95%CI: 1.2935 – 1.9187). Additionally, the MR-Egger, Weighted median, and Weighted mode methods were employed for further analysis (**Table 2**), all indicating a significantly positive correlation between AS and T1DM across the datasets. Although the MR-Egger method suggested a positive trend, significant heterogeneity was identified in the datasets related to T1DM, prompting the use of the IVW method with multiplicative random effects for MR analysis (detailed in **Table 3**). The analysis also highlighted SNPs indicative of horizontal pleiotropy with the outcomes (**Table 4**). Following the identification of these SNPs, they were excluded, and the MR analysis was refined accordingly.

**Table 2:** The Mendelian Randomization analysis between ankylosing spondylitis on diabetes and diabetes-related clinical index based on MR-Egger, Weighted median, and Weighted mode methods.

Exposure	Outcome	Sources	N_SNP	MR-Egger						Weighted median						Weighted mode					
				beta	se	*Pval*	OR	lower95%CI	upper95%CI	beta	se	*Pval*	OR	lower95%CI	upper95%CI	beta	se	*Pval*	OR	lower95%CI	upper95%CI
Ankylosing spondylitis	T1D	ebi-a-GCST005536	17	0.760	0.486	0.139	2.137	0.825	5.536	0.589	0.199	0.003	1.802	1.220	2.663	0.664	0.278	0.030	1.943	1.126	3.350
		ebi-a-GCST010681	20	1.015	0.541	0.077	2.759	0.956	7.966	0.600	0.251	0.017	1.822	1.115	2.977	1.124	0.396	0.011	3.077	1.415	6.689
		ebi-a-GCST90000529	20	1.054	0.478	0.041	2.869	1.124	7.323	0.540	0.214	0.012	1.717	1.129	2.610	0.632	0.226	0.012	1.882	1.207	2.933
		finn-b-T1D_WIDE	20	1.162	0.542	0.046	3.197	1.105	9.249	0.662	0.217	0.002	1.939	1.266	2.970	0.768	0.266	0.009	2.156	1.280	3.631
	T2D	ebi-a-GCST006867	16	0.023	0.064	0.720	1.023	0.904	1.159	0.041	0.045	0.355	1.042	0.955	1.137	0.045	0.046	0.348	1.046	0.955	1.146
		ebi-a-GCST010118	21	0.139	0.062	0.039	1.149	1.017	1.299	0.128	0.041	0.002	1.137	1.049	1.233	0.111	0.039	0.010	1.117	1.034	1.206
		ebi-a-GCST007515	4	-0.032	0.102	0.784	0.969	0.793	1.183	0.039	0.041	0.339	1.040	0.960	1.126	0.038	0.044	0.450	1.039	0.953	1.133
		ebi-a-GCST005413	24	0.202	0.275	0.470	1.224	0.714	2.096	0.167	0.156	0.283	1.182	0.871	1.605	-0.117	0.239	0.629	0.889	0.557	1.421
		ieu-a-23	20	-0.171	0.130	0.204	0.842	0.653	1.087	-0.139	0.085	0.099	0.870	0.737	1.027	-0.139	0.078	0.090	0.870	0.747	1.014
		ieu-a-24	7	-0.238	0.135	0.138	0.788	0.605	1.027	-0.033	0.076	0.662	0.967	0.833	1.123	-0.030	0.072	0.690	0.970	0.842	1.118
		FinnGen_R9_T2D	22	0.020	0.072	0.780	1.021	0.886	1.176	0.020	0.042	0.638	1.020	0.940	1.107	0.016	0.045	0.722	1.016	0.931	1.109
	Fasting insulin	ebi-a-GCST90002238	25	-0.027	0.023	0.250	0.973	0.930	1.018	-0.032	0.013	0.011	0.968	0.944	0.993	-0.029	0.013	0.039	0.972	0.947	0.997
		ieu-b-115	19	-0.012	0.030	0.690	0.988	0.931	1.048	-0.029	0.020	0.143	0.972	0.935	1.010	-0.022	0.020	0.279	0.978	0.941	1.017
		ieu-b-116	5	-0.033	0.019	0.175	0.967	0.932	1.004	-0.021	0.013	0.106	0.979	0.955	1.004	-0.022	0.013	0.171	0.978	0.954	1.004
		ebi-a-GCST000571	19	-0.017	0.037	0.648	0.983	0.915	1.057	-0.040	0.024	0.100	0.961	0.917	1.008	-0.027	0.025	0.296	0.974	0.927	1.022
		ebi-a-GCST007857	4	0.004	0.040	0.934	1.004	0.928	1.086	0.015	0.023	0.533	1.015	0.969	1.062	0.014	0.023	0.578	1.014	0.970	1.061
		ebi-a-GCST008033	25	-0.046	0.079	0.568	0.955	0.818	1.116	-0.043	0.062	0.486	0.958	0.849	1.081	-0.044	0.059	0.461	0.957	0.852	1.074
	Glycated hemoglobin	ebi-a-GCST90002244	22	-0.008	0.013	0.537	0.992	0.966	1.018	-0.006	0.008	0.451	0.994	0.978	1.010	-0.005	0.008	0.508	0.995	0.979	1.010
		ukb-d-30750_irnt	17	0.105	0.053	0.067	1.111	1.001	1.232	0.061	0.024	0.011	1.063	1.014	1.114	0.059	0.027	0.045	1.061	1.006	1.120
	Blood glucose	ebi-a-GCST005186	19	0.019	0.024	0.450	1.019	0.972	1.068	0.024	0.019	0.201	1.024	0.987	1.062	0.021	0.018	0.248	1.022	0.986	1.058
		ebi-a-GCST000568	19	-0.003	0.029	0.928	0.997	0.943	1.055	0.015	0.022	0.483	1.015	0.973	1.060	0.013	0.020	0.532	1.013	0.973	1.054
		ebi-a-GCST007858	4	0.050	0.038	0.317	1.051	0.976	1.132	0.050	0.022	0.025	1.051	1.006	1.097	0.052	0.024	0.116	1.054	1.005	1.104
	Fasting glucose	ebi-a-GCST90002232	24	0.014	0.018	0.428	1.014	0.980	1.050	0.008	0.012	0.476	1.008	0.985	1.032	0.012	0.012	0.299	1.012	0.990	1.036
		ieu-b-114	4	0.012	0.020	0.612	1.012	0.973	1.053	0.014	0.013	0.277	1.014	0.989	1.041	0.014	0.014	0.378	1.015	0.987	1.043
		ieu-b-113	19	0.019	0.024	0.450	1.019	0.972	1.068	0.024	0.019	0.204	1.024	0.987	1.062	0.021	0.017	0.219	1.022	0.989	1.056

T1D, Type 1 diabetes; T2D, Type 2 diabetes.

**Table 3:** The sensitivity analysis between ankylosing spondylitis on diabetes and diabetes-related clinical index by MR-PRESSO, Egger intercept, Q-test, and heterogeneity I2 methods.

Exposure	Outcome	Sources	N_SNP	MR-PRESSO				Egger_intercept	Pval	Q_statistics	Pval	Heterogeneity_I^2%^
				beta	se	*Pval*	GlobalTest_*Pval*					
Ankylosing spondylitis	T1D	ebi-a-GCST005536	17	0.415	0.183	0.037	0.009	-0.014	0.455	32.865	0.008	51
		ebi-a-GCST010681	20	0.573	0.231	0.023	0.001	-0.020	0.377	46.800	0.000	59
		ebi-a-GCST90000529	20	0.474	0.210	0.036	0.001	-0.026	0.196	45.325	0.001	58
		finn-b-T1D_WIDE	20	0.401	0.190	0.049	0.026	-0.028	0.152	32.953	0.024	42
	T2D	ebi-a-GCST006867	16	0.029	0.037	0.451	0.492	0.000	0.917	15.356	0.426	2
		ebi-a-GCST010118	21	0.087	0.037	0.030	0.159	-0.003	0.313	27.718	0.116	28
		ebi-a-GCST007515	4	0.038	0.059	0.565	0.443	0.008	0.480	6.613	0.085	55
		ebi-a-GCST005413	24	0.170	0.114	0.149	0.091	-0.001	0.899	32.034	0.099	28
		ieu-a-23	20	-0.083	0.076	0.287	0.170	0.006	0.412	26.344	0.121	28
		ieu-a-24	7	0.042	0.136	0.766	0.242	0.029	0.036	20.529	0.002	71
		FinnGen_R9_T2D	22	0.034	0.041	0.418	0.017	0.001	0.814	39.234	0.009	46
	Fasting insulin	ebi-a-GCST90002238	25	-0.023	0.013	0.092	0.004	0.000	0.829	48.580	0.002	51
		ieu-b-115	19	-0.030	0.018	0.115	0.102	-0.001	0.473	26.548	0.088	32
		ieu-b-116	5	-0.016	0.011	0.213	0.485	0.002	0.295	2.795	0.593	0
		ebi-a-GCST000571	19	-0.027	0.022	0.222	0.143	-0.001	0.731	25.781	0.105	30
		ebi-a-GCST007857	4	0.023	0.016	0.230	0.542	0.002	0.610	1.412	0.703	0
		ebi-a-GCST008033	25	-0.076	0.046	0.108	0.419	-0.002	0.640	25.217	0.394	5
	Glycated hemoglobin	ebi-a-GCST90002244	22	0.002	0.008	0.839	0.060	0.001	0.362	34.267	0.034	39
		ukb-d-30750_irnt	17	0.057	0.021	0.017	0.046	-0.002	0.338	27.173	0.040	41
	Blood glucose	ebi-a-GCST005186	19	0.025	0.014	0.094	0.624	0.000	0.745	16.837	0.534	0
		ebi-a-GCST000568	19	0.018	0.017	0.304	0.621	0.001	0.383	16.933	0.528	0
		ebi-a-GCST007858	4	0.051	0.015	0.047	0.719	0.000	0.986	1.548	0.671	0
	Fasting glucose	ebi-a-GCST90002232	24	0.014	0.010	0.188	0.112	0.000	0.963	33.185	0.078	31
		ieu-b-114	4	0.015	0.008	0.159	0.881	0.000	0.885	1.141	0.767	0
		ieu-b-113	19	0.025	0.014	0.094	0.643	0.000	0.745	16.837	0.534	0

T1D, Type 1 diabetes; T2D, Type 2 diabetes.

**Table 4:** Genetic instrumental variables detected by the MR-PRESSO method to show pleiotropy with ankylosing spondylitis on type 2 diabetes and glycated hemoglobin.

Explore	Outcome	Dataset	SNP	Chr	Pos	EA	NEA	Beta	Se	Pval
Ankylosing spondylitis	Type 2 diabetes	ebi-a-GCST005413	rs2596501	6	31321211	T	C	-0.152336	0.00419401	1E-200
	Type 2 diabetes	ebi-a-GCST010118	rs11190133	10	101278725	T	C	-0.0338671	0.00449401	4.84E-14
	Type 2 diabetes	finngen_R9_T2D	rs4129267	1	154426264	T	C	-0.0307685	0.00422605	3.32E-13
	Type 2 diabetes	ieu-a-26	rs1128905	9	139253839	C	T	-0.0237165	0.00409459	6.95E-09
	Type 2 diabetes	ieu-a-976	rs1128905	9	139253839	C	T	-0.0237165	0.00409459	6.95E-09
	Glycated hemoglobin	ukb-d-30750_irnt	rs1860545	12	6446777	A	G	-0.027474	0.00435378	2.78E-10
	Glycated hemoglobin	ukb-d-30750_irnt	rs2596501	6	31321211	T	C	-0.152336	0.00419401	1E-200
	Glycated hemoglobin	ukb-d-30750_raw	rs11065898	12	111862575	T	C	0.0262524	0.00480641	4.71E-08
	Glycated hemoglobin	ukb-d-30750_raw	rs9901869	17	45575206	A	G	0.0319036	0.00408853	6.04E-15

### Effect of AS on T2DM

The analysis incorporated seven GWAS summary datasets related to type 2 diabetes, comprising 319,375 patients and 1,765,138 controls. **Figure 2** demonstrates that a significantly positive correlation between AS and T2DM was identified in one of the datasets, with an odds ratio (OR) of 1.0910 and a 95% confidence interval (CI) from 1.0144 to 1.1734. Meanwhile, five datasets displayed a positive association that did not reach statistical significance. Notably, one dataset revealed a non-significant negative correlation between AS and T2DM (OR= 0.92, 95%CI: 0.7928 – 1.0677). Given the lack of heterogeneity among the datasets (I2 = 0, p=0.49), a fixed-effect model was applied to aggregate the results. The pooled analysis from the meta-analysis showed a significantly positive link between AS and T2DM (OR=1.0519, 95%CI: 1.0059 – 1.1001). Subsequent heterogeneity testing indicated the presence of significant heterogeneity in certain datasets. Consequently, the IVW method with multiplicative random effects was employed for the MR analysis. Additionally, MR-PRESSO analysis was conducted to investigate any SNPs indicative of horizontal pleiotropy with the outcomes, which were then excluded from further analysis as detailed in the subsequent study.

### Effect of AS on blood glucose, fasting glucose, and glycated hemoglobin

**Figure 3:**
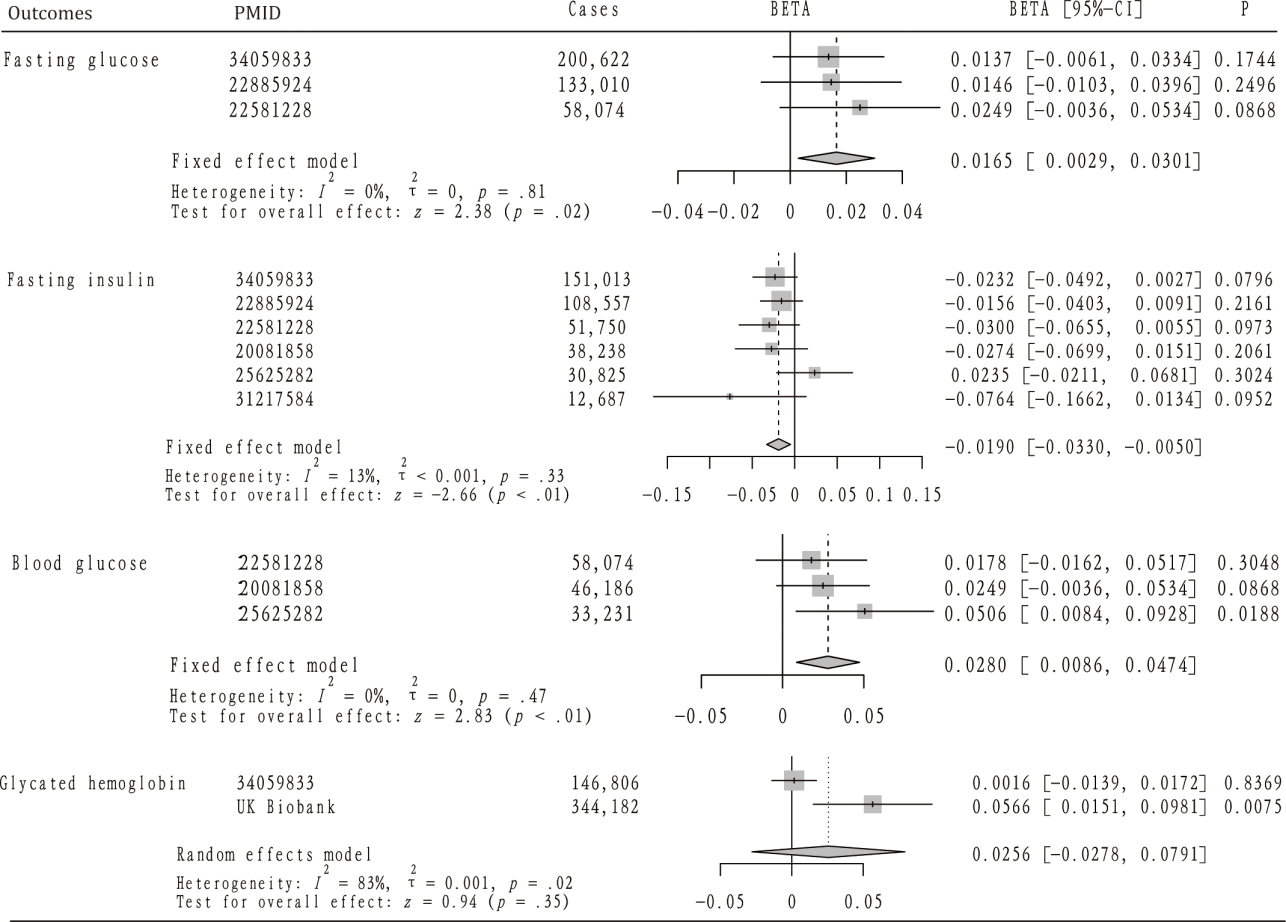
The mendelian randomization analysis and meta-analysis about the casual effect of ankylosing spondylitis on diabetes-related traits.

Our analysis further explored the causal relationship between AS and levels of blood glucose and fasting glucose. According to **Figure 3**, the analysis included three GWAS summary datasets related to blood glucose, encompassing a total of 137,491 participants. Within these datasets, a significantly positive correlation with blood glucose was noted in one dataset (beta = 0.0506, 95%CI: 0.0084 – 0.0928). The remaining two datasets suggested a positive relationship between AS and blood glucose, although these findings did not achieve statistical significance. Owing to the lack of heterogeneity among the datasets, a fixed-effect model was applied to consolidate the MR analysis results. This pooled analysis revealed a significant positive impact of AS on blood glucose levels (beta = 0.0280, 95% CI: 0.0086 – 0.0470). Furthermore, alternative analytical approaches including MR-Egger, Weighted median, and Weighted mode methods did not identify a significant link between AS and blood glucose. Heterogeneity assessments also confirmed the absence of heterogeneity across the studies (**Table 3**).

**Figure 3** also highlights the inclusion of three GWAS summary datasets related to fasting glucose for analysis, involving a total of 391,706 participants. Across all datasets, a positive correlation between AS and fasting glucose levels was noted, although these observations did not reach statistical significance. Given the uniformity of the MR analysis results, indicating no heterogeneity, a fixed-effect model was implemented for the meta-analysis. This analysis revealed that AS has a modest but positive impact on fasting glucose levels (OR=0.0165, 95%CI: 0.0029 – 0.0301). Furthermore, while not reaching statistical significance, MR analyses employing MR-Egger, Weighted median, and Weighted mode methodologies similarly suggested a positive relationship between AS and fasting glucose. Heterogeneity tests confirmed the lack of significant heterogeneity (**Table 3**), and MR-PRESSO analysis did not identify any SNPs indicative of horizontal pleiotropy with the outcomes. In addition, two datasets were analyzed concerning glycated hemoglobin, totaling 490,988 participants. Analysis of the UK Biobank (UKB) dataset indicated a significant increase in glycated hemoglobin levels attributable to AS (beta = 0.0566, 95%CI: 0.0151 – 0.0981), a finding not replicated in the second dataset. Due to detected heterogeneity, a random-effects model was utilized to aggregate MR analysis results, which did not establish a significant overall association. The presence of heterogeneity prompted the use of the IVW method with multiplicative random effects for further analysis. Additionally, any SNPs identified as exhibiting horizontal pleiotropy with outcomes were excluded from the MR analysis (**Table 4**).

### Effect of AS on fasting insulin

**Figure 3** details the inclusion of six datasets for analyzing the potential causal relationship between AS and fasting insulin levels, with a collective participation of 393,070 individuals. Among these, a single dataset indicated a positive correlation between AS and fasting insulin, though this did not achieve statistical significance. Conversely, the remaining five datasets exhibited a negative correlation between AS and fasting insulin, again without reaching statistical significance. Owing to the consistent results across the datasets, indicating no significant heterogeneity, a fixed-effect model was employed for the meta-analysis. This analysis suggested that AS might significantly reduce fasting insulin levels (OR=-0.0190, 95%CI: -0.1662, -0.0134). Significant heterogeneity was observed in only one dataset, leading to the application of the IVW method with multiplicative random effects for further MR analysis. The MR-PRESSO analysis did not identify any SNPs indicative of horizontal pleiotropy with respect to fasting insulin levels. Scatter plots illustrating the impact of ankylosing spondylitis on diabetes and its related traits can be found in **Supplementary Figures S1**, **2**.

## Discussion

Our analysis revealed a significant association between ankylosing spondylitis (AS) and an increased risk of type 1 diabetes mellitus (T1DM) and type 2 diabetes mellitus (T2DM). AS was linked to elevated fasting glucose and blood glucose levels, along with a decrease in fasting insulin levels. The study identified a causal relationship between AS and T2DM, as well as an impact on glucose metabolism traits.

Spondyloarthritis (SpA) encompasses a group of related but clinically varied diseases (33). Ankylosing spondylitis (AS) emerges as the most common and severe form of SpA, predominantly targeting the axial skeleton, especially the sacroiliac joints (4). Characterized by inflammatory back pain, AS leads to significant discomfort and functional impairment in the spine and sacroiliac joints, thereby diminishing the quality of life for those affected and imposing a considerable societal and individual burden (34). AS is a condition that should be on the radar for diagnosis among young people presenting with back pain, particularly in young men (35). The early detection of AS poses a challenge but is of paramount importance. The disease is a complex immune-mediated condition with a pathophysiology that remains largely elusive (7). Historical research dating back to 1973 has established a connection between the inheritance of the HLA-B27 allele and AS (36). In recent years, the IL-17A/IL-23 inflammatory axis has gained significant attention (37). Despite the complexity of its etiology, autoimmune mechanisms are believed to play a critical role in the development of AS.

Diabetes can be broadly categorized into the following main types: (1) Type 1 diabetes, (2) Type 2 diabetes, (3) Gestational diabetes mellitus (GDM), and (4) Specific types of diabetes resulting from other causes (38). T1DM is recognized as an autoimmune condition that necessitates lifelong insulin therapy upon diagnosis (39), and it predominantly affects children and adolescents (40). The underlying causes of T1DM are multifaceted and not fully understood (41), with prevailing research indicating that the disease primarily stems from the immune-mediated destruction of insulin-producing β-cells in the pancreas (42). The onset of diabetes is thought to result from a complex interaction of environmental factors, microbiota, genetics, metabolism, and immune responses that differ among individuals (43). Recent studies have pinpointed several genes within the MHC as key loci associated with susceptibility to diabetes (44), highlighting the role of immune mechanisms in the pathogenesis of T1DM.

In recent years, research interest in autoimmune diseases has surged, with both ankylosing spondylitis (AS) and diabetes being recognized within this category. This has led to a growing curiosity about the potential connection between these two conditions. Numerous studies suggest that individuals with AS are at an increased risk of developing diabetes. For instance, research indicates that people diagnosed with AS are more prone to diabetes than those without the condition (45). Additionally, the incidence of diabetes mellitus in the AS population was found to be 1.21 times higher compared to those without AS (19). Specifically, Chen et al. found that AS was associated with an increased risk of type 2 diabetes among the Asian population, with the AS group experiencing a 1.17 times higher incidence of T2DM compared to the non-AS group (20). A cross-sectional study also revealed that 13.64% of AS patients were diagnosed with diabetes mellitus (46). However, it’s important to note that these studies primarily establish an association rather than a direct causative link between AS and diabetes mellitus.

Our research delves into the relationship between AS and diabetes mellitus by employing two-sample MR analysis. Our findings corroborate previous observational studies by demonstrating that AS is associated with an increased risk of both T1DM and T2DM. Additionally, our study uncovers that AS contributes to elevated fasting blood glucose levels and reduced insulin levels. Notably, our analysis suggests an increase in HBA1c levels associated with AS, although this finding did not reach statistical significance. Fasting glucose, fasting insulin, blood glucose, and glycated hemoglobin may be key biomarkers that may reveal important relationships with AS. Elevated fasting glucose and blood glucose levels in AS patients can indicate insulin resistance and impaired glucose metabolism, linking AS to an increased risk of type 2 diabetes. Similarly, altered fasting insulin levels may reflect the body’s response to inflammation associated with AS, potentially contributing to metabolic disturbances. HbA1c serves as a measure of average blood glucose over time; higher levels in AS patients could signify chronic hyperglycemia and a greater risk for diabetes. Monitoring these metabolic parameters is crucial for managing the health of individuals with AS and addressing potential metabolic complications. To the best of our knowledge, this is the first study to establish a causal link between AS and disruptions in glucose metabolism leading to diabetes mellitus, advancing beyond the mere observational associations reported by prior research. However, the underlying mechanisms of interaction between AS and diabetes mellitus demand further exploration. I propose several hypotheses centered around immunological mechanisms to guide future inquiries into this relationship.

The programmed death 1 (PD-1) pathway, including its ligands PD-L1 and PD-L2, is crucial for the inhibitory signaling of T cells. Disruptions in this pathway can lead to autoimmune diseases, such as AS and T1DM (47). For instance, research indicates that AS patients exhibit notably lower levels of PD-1+CD3+ and PD-1+CD4+ T cells (48). In animal models, specifically the L1C transgenic NOD mice that express higher levels of PD-L1, a reduced occurrence of spontaneous diabetes has been observed, suggesting a protective role of PD-L1 against diabetes (49). Dysfunctions in PD-1 and PD-L1 have been associated with increased infiltration of Th1 cells into the pancreatic islets (50), exacerbating the risk of diabetes by promoting a Th1-dominated (IFN-gamma) pancreatic environment (51). Additionally, a meta-analysis highlighted the significance of PD1.5 and PD1.9 polymorphisms in both AS and T1DM (52). Thus, our investigation supports the hypothesis that PD-1 pathway abnormalities contribute to the development of both AS and type 1 diabetes, revealing a potential immunological link between these conditions.

Osteopontin (OPN) serves as a crucial regulator within both innate and adaptive immune responses, acting as a versatile cytokine and adhesion molecule (53). Studies have shown that individuals with ankylosing spondylitis (AS) exhibit elevated levels of OPN compared to healthy controls (54). Furthermore, genetic variations in the OPN gene have been linked to an increased risk of AS among the Han Chinese population (55). Similarly, elevated serum levels of OPN have been observed in adults diagnosed with T1DM (56). While the specific contributions of OPN to the pathophysiology of AS and T1D warrant further exploration, its role has been more thoroughly investigated in other autoimmune conditions, particularly rheumatoid arthritis. This indicates that OPN is a significant factor in the landscape of autoimmune diseases, although the exact mechanisms through which it operates remain to be fully elucidated.

Reactive oxygen species (ROS), produced as byproducts of metabolic activities, are implicated in numerous biological processes, such as inflammation, cancer, and aging (57). There is a growing body of research focusing on the relationship between AS and oxidative stress. Specifically, the underlying mechanisms of AS might involve an elevation in oxidative agents coupled with a reduction in the body’s antioxidant defense capabilities (58). Markers of oxidative stress have been found to be higher in patients with AS (59). There is evidence suggesting that oxidative stress contributes to the development of insulin resistance (60, 61). Insulin resistance is a condition where the body’s cells become less responsive to insulin, impacting the uptake, metabolism, or storage of glucose, and has been associated with AS (62). Previous research indicates that levels of tumor necrosis factor (TNF), interleukin (IL)-1, and IL-6 are elevated in AS and contribute to the onset of insulin resistance (63). Given that insulin resistance is a key factor in the development of diabetes, it is plausible to consider whether oxidative stress, along with the elevated release of IL-1, IL-6, and TNF in AS, could lead to insulin resistance, thereby playing a role in the development of diabetes.

TNF is a critical factor in the pathogenesis of ankylosing spondylitis (AS), leading to the development of TNF-targeting therapies for its treatment (64). These TNF pathways are central to the immune-mediated inflammatory response seen in AS (65). However, treatments that target TNF have been observed to affect metabolic processes, including those involved in glucose metabolism (66). Research by Sidiropoulos indicated that anti-TNF therapy might promote insulin resistance and reduce the activity of glucose transporter protein 4 (67). Conversely, anti-TNF treatment has been associated with a decrease in plasma glucose levels through enhanced insulin sensitivity (68). Furthermore, the risk of diabetes mellitus in AS patients appears to be lower when anti-TNF therapy is combined with hydroxychloroquine (HCQ) (69). This body of research underscores the complex impact of anti-TNF treatments on glucose metabolism, suggesting a potential link between AS management strategies and the development of diabetes.

Recent research has placed the gut microbiome at the forefront of investigations into human autoimmune diseases. The ‘epithelial barrier theory,’ as proposed by Akdis, suggests that microbial dysbiosis and translocation can activate the immune system, leading to inflammatory conditions (70). Metagenomic studies have identified significant changes in the pro-inflammatory gut microbiota of individuals with ankylosing spondylitis (AS), indicating an alteration in the gut microbiota of patients who have not undergone treatment (71). These patients also exhibit dysbiosis, which affects intestinal epithelial and vascular barriers, and an increase in blood zonulin levels, suggesting compromised intestinal barrier function (63). It is estimated that intestinal mucosal inflammation occurs in approximately 70% of individuals with AS (72). The influence of HLA-B27 on the gut microbiota composition in AS has been noted as significant, echoing findings in type 1 diabetes, where distinct microbial compositions have been observed compared to healthy individuals (73). Previous research has identified significant variations in the microbial composition of people with type 1 diabetes compared to healthy controls (74). In cases of diabetes, a breach in the intestinal barrier’s integrity has been linked to T cell-mediated autoimmunity against islet cells (75). Specifically, enteric bacterial infections compromising the intestinal barrier can activate diabetogenic CD8(+) T cells, leading to insulitis (76). The Zonulin family peptide, a key regulator of intestinal tight junctions, is implicated in the development of a permeable intestinal barrier, dysbiosis, and inflammation (77). This evidence underscores the potential role of gut microbiome imbalance in the development of diabetes in individuals with AS.

Our study offers several advantages. Notably, it is among the first to leverage MR analysis to elucidate a causal link between AS and diabetes, with large-scale GWAS providing a refined understanding of their association. The use of MR analysis helps circumvent common pitfalls such as confounders and reverse causation, enhancing the reliability of our findings. However, the study is not without its limitations. A significant constraint is the lack of sex-specific analysis, which is critical given the varied prevalence of autoimmune diseases between genders. Future studies incorporating gender-specific MR analyses could offer more nuanced insights. Additionally, our research focused on European populations, limited by the availability of GWAS data, making our findings less generalizable to other ethnicities. Despite using various methods to assess heterogeneity, the possibility of residual heterogeneity remains. MR heterogeneity, stemming from factors like population differences and study design, must be addressed to improve result accuracy and credibility. Consequently, we cannot definitively conclude whether AS has a direct or indirect association with T1DM or T2DM. Further multivariable MR and mediation analyses are needed to clarify these relationships. In summary, our results lend support to a possible causal relationship between AS and diabetes mellitus. While MR analyses mitigate certain biases and errors, the limitations noted necessitate further investigations into the underlying mechanisms by which AS may contribute to the development of diabetes.

## Conclusion

This study discovered, via MR analysis, that AS elevates the risk of both T1DM and T2DM. Additionally, our findings indicate that AS is associated with higher fasting and blood glucose levels, alongside a reduction in fasting insulin. Collectively, these outcomes highlight the detrimental impact of AS on diabetes development, underscoring the critical role of autoimmune disorders in diabetes pathogenesis. This research significantly contributes to our understanding of the immunological mechanisms underlying diabetes development.

## Data Availability

The original contributions presented in the study are included in the article/**Supplementary Material**. Further inquiries can be directed to the corresponding authors.

## References

[1] Mauro D Thomas R Guggino G Lories R Brown MA Ciccia F. Ankylosing spondylitis: an autoimmune or autoinflammatory disease? Nat Rev Rheumatol. (2021) 17:387–404. doi: 10.1038/s41584-021-00625-y 34113018

[2] Ritchlin C Adamopoulos IE. Axial spondyloarthritis: new advances in diagnosis and management. Bmj. (2021) 372:m4447. doi: 10.1136/bmj.m4447 33397652

[3] Sieper J Braun J Dougados M Baeten D. Axial spondyloarthritis. Nat Rev Dis Primers. (2015) 1:15013. doi: 10.1038/nrdp.2015.13 27188328

[4] Smith JA. Update on ankylosing spondylitis: current concepts in pathogenesis. Curr Allergy Asthma Rep. (2015) 15:489. doi: 10.1007/s11882-014-0489-6 25447326

[5] Landi M Maldonado-Ficco H Perez-Alamino R Maldonado-Cocco JA Citera G Arturi P. Gender differences among patients with primary ankylosing spondylitis and spondylitis associated with psoriasis and inflammatory bowel disease in an iberoamerican spondyloarthritis cohort. Med (Baltimore). (2016) 95:e5652. doi: 10.1097/MD.0000000000005652 PMC5181818 28002334

[6] Yang H Chen Y Xu W Shao M Deng J Xu S. Epigenetics of ankylosing spondylitis: Recent developments. Int J Rheum Dis. (2021) 24:487–93. doi: 10.1111/1756-185X.14080 33608999

[7] Simone D Al Mossawi MH Bowness P. Progress in our understanding of the pathogenesis of ankylosing spondylitis. Rheumatol (Oxford). (2018) 57:vi4–9. doi: 10.1093/rheumatology/key001 PMC6238220 30445483

[8] Chen CW Wei JC Gu J Yu D. Editorial: advances in pathogenesis, etiology, and therapies for ankylosing spondylitis. Front Immunol. (2021) 12:822582. doi: 10.3389/fimmu.2021.822582 35003143 PMC8732985

[9] Zhu W He X Cheng K Zhang L Chen D Wang X. Ankylosing spondylitis: etiology, pathogenesis, and treatments. Bone Res. (2019) 7:22. doi: 10.1038/s41413-019-0057-8 31666997 PMC6804882

[10] Xu GH Lin J Chen WQ. Concurrent ankylosing spondylitis and myelodysplastic syndrome: A case report. World J Clin cases. (2022) 10:1929–36. doi: 10.12998/wjcc.v10.i6.1929 PMC8891766 35317144

[11] Rezaiemanesh A Abdolmaleki M Abdolmohammadi K Aghaei H Pakdel FD Fatahi Y. Immune cells involved in the pathogenesis of ankylosing spondylitis. BioMed Pharmacother. (2018) 100:198–204. doi: 10.1016/j.biopha.2018.01.108 29428668

[12] Ou J Xiao M Huang Y Tu L Chen Z Cao S. Serum metabolomics signatures associated with ankylosing spondylitis and TNF inhibitor therapy. Front Immunol. (2021) 12:630791. doi: 10.3389/fimmu.2021.630791 33679777 PMC7933516

[13] Ogurtsova K da Rocha Fernandes JD Huang Y Linnenkamp U Guariguata L Cho NH. IDF Diabetes Atlas: Global estimates for the prevalence of diabetes for 2015 and 2040. Diabetes Res Clin Pract. (2017) 128:40–50. doi: 10.1016/j.diabres.2017.03.024 28437734

[14] Katsarou A Gudbjörnsdottir S Rawshani A Dabelea D Bonifacio E Anderson BJ. Type 1 diabetes mellitus. Nat Rev Dis Primers. (2017) 3:17016. doi: 10.1038/nrdp.2017.16 28358037

[15] Chatterjee S Khunti K Davies MJ. Type 2 diabetes. Lancet. (2017) 389:2239–51. doi: 10.1016/S0140-6736(17)30058-2 28190580

[16] Donath MY Dinarello CA Mandrup-Poulsen T. Targeting innate immune mediators in type 1 and type 2 diabetes. Nat Rev Immunol. (2019) 19:734–46. doi: 10.1038/s41577-019-0213-9 31501536

[17] Dror E Dalmas E Meier DT Wueest S Thévenet J Thienel C. Postprandial macrophage-derived IL-1β stimulates insulin, and both synergistically promote glucose disposal and inflammation. Nat Immunol. (2017) 18:283–92. doi: 10.1038/ni.3659 28092375

[18] Biondi B Kahaly GJ Robertson RP. Thyroid dysfunction and diabetes mellitus: two closely associated disorders. Endocr Rev. (2019) 40:789–824. doi: 10.1210/er.2018-00163 30649221 PMC6507635

[19] Liao KF Kuo YH Lai SW. Diabetes mellitus in ankylosing spondylitis. Ann Rheum Dis. (2021) 80:e134. doi: 10.1136/annrheumdis-2019-216221 31492707

[20] Chen HH Yeh SY Chen HY Lin CL Sung FC Kao CH. Ankylosing spondylitis and other inflammatory spondyloarthritis increase the risk of developing type 2 diabetes in an Asian population. Rheumatol Int. (2014) 34:265–70. doi: 10.1007/s00296-013-2927-5 24362789

[21] Sekula P Del Greco MF Pattaro C Köttgen A. Mendelian randomization as an approach to assess causality using observational data. J Am Soc Nephrol. (2016) 27:3253–65. doi: 10.1681/ASN.2016010098 PMC5084898 27486138

[22] Hemani G Zheng J Elsworth B Wade KH Haberland V Baird D. The MR-Base platform supports systematic causal inference across the human phenome. Elife. (2018) 7:e34408. doi: 10.7554/eLife.34408 29846171 PMC5976434

[23] Davey Smith G Hemani G. Mendelian randomization: genetic anchors for causal inference in epidemiological studies. Hum Mol Genet. (2014) 23:R89–98. doi: 10.1093/hmg/ddu328 PMC4170722 25064373

[24] Su Q Jin C Yang Y Wang J Wang J Zeng H. Association between autoimmune diseases and sarcopenia: A two-sample mendelian randomization study. Clin Epidemiol. (2023) 15:901–10. doi: 10.2147/CLEP.S416778 PMC10464831 37650009

[25] Sun D Ma R Wang J Wang Y Ye Q. The causal relationship between sarcoidosis and autoimmune diseases: a bidirectional Mendelian randomization study in FinnGen. Front Immunol. (2024) 15:1325127. doi: 10.3389/fimmu.2024.1325127 38711527 PMC11070530

[26] Su Y Zhang Y Chai Y Xu J. Autoimmune diseases and their genetic link to bronchiectasis: insights from a genetic correlation and Mendelian randomization study. Front Immunol. (2024) 15:1343480. doi: 10.3389/fimmu.2024.1343480 38660310 PMC11039849

[27] Li S Yuan S Schooling CM Larsson SC. A Mendelian randomization study of genetic predisposition to autoimmune diseases and COVID-19. Sci Rep. (2022) 12:17703. doi: 10.1038/s41598-022-22711-1 36271292 PMC9587049

[28] Zhang Y Liu J Wang C Liu J Lu W. Toll-like receptors gene polymorphisms in autoimmune disease. Front Immunol. (2021) 12:672346. doi: 10.3389/fimmu.2021.672346 33981318 PMC8107678

[29] Cortes A Hadler J Pointon JP Robinson PC Karaderi T Leo P. Identification of multiple risk variants for ankylosing spondylitis through high-density genotyping of immune-related loci. Nat Genet. (2013) 45:730–8. doi: 10.1038/ng.2667 PMC3757343 23749187

[30] Cheng Q Yang Y Shi X Yeung KF Yang C Peng H. MR-LDP: a two-sample Mendelian randomization for GWAS summary statistics accounting for linkage disequilibrium and horizontal pleiotropy. NAR Genom Bioinform. (2020) 2:lqaa028. doi: 10.1093/nargab/lqaa028 33575584 PMC7671398

[31] Pierce BL Ahsan H Vanderweele TJ. Power and instrument strength requirements for Mendelian randomization studies using multiple genetic variants. Int J Epidemiol. (2011) 40:740–52. doi: 10.1093/ije/dyq151 PMC3147064 20813862

[32] Adu Y Ring D Teunis T. Randomized controlled trials studying nonoperative treatments of osteoarthritis often use misleading and uninformative control groups: A systematic review. Clin Orthop Relat Res. (2024). doi: 10.1097/CORR.0000000000003273 PMC11936557 39453403

[33] Dougados M Baeten D. Spondyloarthritis. Lancet. (2011) 377:2127–37. doi: 10.1016/S0140-6736(11)60071-8 21684383

[34] Burgos-Varga R Wei JC Rahman MU Akkoc N Haq SA Hammoudeh M. The prevalence and clinical characteristics of nonradiographic axial spondyloarthritis among patients with inflammatory back pain in rheumatology practices: a multinational, multicenter study. Arthritis Res Ther. (2016) 18:132. doi: 10.1186/s13075-016-1027-9 27267875 PMC4896040

[35] Escalante A. Ankylosing spondylitis. A common cause of low back pain. Postgrad Med. (1993) 94:153–60. doi: 10.1080/00325481.1993.11945685 8321769

[36] Schlosstein L Terasaki PI Bluestone R Pearson CM. High association of an HL-A antigen, W27, with ankylosing spondylitis. N Engl J Med. (1973) 288:704–6. doi: 10.1056/NEJM197304052881403 4688372

[37] McGeachy MJ Chen Y Tato CM Laurence A Joyce-Shaikh B Blumenschein WM. The interleukin 23 receptor is essential for the terminal differentiation of interleukin 17-producing effector T helper cells in vivo. Nat Immunol. (2009) 10:314–24. doi: 10.1038/ni.1698 PMC2945605 19182808

[38] (2) Classification and diagnosis of diabetes. Diabetes Care. (2015) 38 Suppl:S8–s16. doi: 10.2337/dc15-S005 25537714

[39] Bluestone JA Herold K Eisenbarth G. Genetics, pathogenesis and clinical interventions in type 1 diabetes. Nature. (2010) 464:1293–300. doi: 10.1038/nature08933 PMC4959889 20432533

[40] Harjutsalo V Sjöberg L Tuomilehto J. Time trends in the incidence of type 1 diabetes in Finnish children: a cohort study. Lancet. (2008) 371:1777–82. doi: 10.1016/S0140-6736(08)60765-5 18502302

[41] Ni Q Pham NB Meng WS Zhu G Chen X. Advances in immunotherapy of type I diabetes. Adv Drug Deliv Rev. (2019) 139:83–91. doi: 10.1016/j.addr.2018.12.003 30528629

[42] Anderson MS Bluestone JA. The NOD mouse: a model of immune dysregulation. Annu Rev Immunol. (2005) 23:447–85. doi: 10.1146/annurev.immunol.23.021704.115643 15771578

[43] DiMeglio LA Evans-Molina C Oram RA. Type 1 diabetes. Lancet. (2018) 391:2449–62. doi: 10.1016/S0140-6736(18)31320-5 PMC6661119 29916386

[44] Mordes JP Bortell R Doukas J Rigby M Whalen B Zipris D. The BB/Wor rat and the balance hypothesis of autoimmunity. Diabetes Metab Rev. (1996) 12:103–9. doi: 10.1002/(SICI)1099-0895(199607)12:2<103::AID-DMR157>3.0.CO;2-2 8877279

[45] Wang S Tsou HK Chiou JY Wang YH Zhang Z Wei JC. Increased risk of inflammatory bowel disease among patients with ankylosing spondylitis: A 13-year population-based cohort study. Front Immunol. (2020) 11:578732. doi: 10.3389/fimmu.2020.578732 33123163 PMC7567031

[46] Kang JH Chen YH Lin HC. Comorbidity profiles among patients with ankylosing spondylitis: a nationwide population-based study. Ann Rheum Dis. (2010) 69:1165–8. doi: 10.1136/ard.2009.116178 20375121

[47] Zamani MR Aslani S Salmaninejad A Javan MR Rezaei N. PD-1/PD-L and autoimmunity: A growing relationship. Cell Immunol. (2016) 310:27–41. doi: 10.1016/j.cellimm.2016.09.009 27660198

[48] Chen MH Chen WS Lee HT Tsai CY Chou CT. Inverse correlation of programmed death 1 (PD-1) expression in T cells to the spinal radiologic changes in Taiwanese patients with ankylosing spondylitis. Clin Rheumatol. (2011) 30:1181–7. doi: 10.1007/s10067-011-1721-6 21547439

[49] Wang CJ Chou FC Chu CH Wu JC Lin SH Chang DM. Protective role of programmed death 1 ligand 1 (PD-L1)in nonobese diabetic mice: the paradox in transgenic models. Diabetes. (2008) 57:1861–9. doi: 10.2337/db07-1260 PMC2453619 18420489

[50] Wang J Yoshida T Nakaki F Hiai H Okazaki T Honjo T. Establishment of NOD-Pdcd1-/- mice as an efficient animal model of type I diabetes. Proc Natl Acad Sci USA. (2005) 102:11823–8. doi: 10.1073/pnas.0505497102 PMC1188011 16087865

[51] Hill NJ Van Gunst K Sarvetnick N. Th1 and Th2 pancreatic inflammation differentially affects homing of islet-reactive CD4 cells in nonobese diabetic mice. J Immunol. (2003) 170:1649–58. doi: 10.4049/jimmunol.170.4.1649 12574327

[52] Lee YH Bae SC Kim JH Song GG. Meta-analysis of genetic polymorphisms in programmed cell death 1. Associations with rheumatoid arthritis, ankylosing spondylitis, and type 1 diabetes susceptibility. Z Rheumatol. (2015) 74:230–9. doi: 10.1007/s00393-014-1415-y 24942602

[53] Xu C Wu Y Liu N. Osteopontin in autoimmune disorders: current knowledge and future perspective. Inflammopharmacology. (2022) 30:385–96. doi: 10.1007/s10787-022-00932-0 35235108

[54] Choi ST Kim JH Kang EJ Lee SW Park MC Park YB. Osteopontin might be involved in bone remodeling rather than in inflammation in ankylosing spondylitis. Rheumatol (Oxford). (2008) 47:1775–9. doi: 10.1093/rheumatology/ken385 18854347

[55] Li J Cai Y Wang Z Deng A Yang G. Polymorphisms in the osteopontin are associated with susceptibility to ankylosing spondylitis in a han chinese population. BioMed Res Int 2018. (2018) p:3458439. doi: 10.1155/2018/3458439 PMC5822803 29581970

[56] Barchetta I Alessandri C Bertoccini L Cimini FA Taverniti L Di Franco M. Increased circulating osteopontin levels in adult patients with type 1 diabetes mellitus and association with dysmetabolic profile. Eur J Endocrinol. (2016) 174:187–92. doi: 10.1530/EJE-15-0791 26578639

[57] Kawahito S Kitahata H Oshita S. Problems associated with glucose toxicity: role of hyperglycemia-induced oxidative stress. World J Gastroenterol. (2009) 15:4137–42. doi: 10.3748/wjg.15.4137 PMC2738809 19725147

[58] Karakoc M Altindag O Keles H Soran N Selek S. Serum oxidative-antioxidative status in patients with ankylosing spondilitis. Rheumatol Int. (2007) 27:1131–4. doi: 10.1007/s00296-007-0352-3 17443328

[59] Ye G Xie Z Zeng H Wang P Li J Zheng G. Oxidative stress-mediated mitochondrial dysfunction facilitates mesenchymal stem cell senescence in ankylosing spondylitis. Cell Death Dis. (2020) 11:775. doi: 10.1038/s41419-020-02993-x 32943613 PMC7498590

[60] Rudich A Tirosh A Potashnik R Hemi R Kanety H Bashan N. Prolonged oxidative stress impairs insulin-induced GLUT4 translocation in 3T3-L1 adipocytes. Diabetes. (1998) 47:1562–9. doi: 10.2337/diabetes.47.10.1562 9753293

[61] Dokken BB Saengsirisuwan V Kim JS Teachey MK Henriksen EJ. Oxidative stress-induced insulin resistance in rat skeletal muscle: role of glycogen synthase kinase-3. Am J Physiol Endocrinol Metab. (2008) 294:E615–21. doi: 10.1152/ajpendo.00578.2007 18089761

[62] Khodabandehloo H Gorgani-Firuzjaee S Panahi G Meshkani R. Molecular and cellular mechanisms linking inflammation to insulin resistance and β-cell dysfunction. Transl Res. (2016) 167:228–56. doi: 10.1016/j.trsl.2015.08.011 26408801

[63] Wang CR Tsai HW. Anti- and non-tumor necrosis factor-α-targeted therapies effects on insulin resistance in rheumatoid arthritis, psoriatic arthritis and ankylosing spondylitis. World J Diabetes. (2021) 12:238–60. doi: 10.4239/wjd.v12.i3.238 PMC7958474 33758645

[64] Li J Zhang Z Wu X Zhou J Meng D Zhu P. Risk of adverse events after anti-TNF treatment for inflammatory rheumatological disease. A meta-analysis. Front Pharmacol. (2021) 12:746396. doi: 10.3389/fphar.2021.746396 34790122 PMC8591221

[65] Brown MA Kenna T Wordsworth BP. Genetics of ankylosing spondylitis—insights into pathogenesis. Nat Rev Rheumatol. (2016) 12:81–91. doi: 10.1038/nrrheum.2015.133 26439405

[66] da Silva BS Bonfá E de Moraes JC Saad CG Ribeiro AC Gonçalves CR. Effects of anti-TNF therapy on glucose metabolism in patients with ankylosing spondylitis, psoriatic arthritis or juvenile idiopathic arthritis. Biologicals. (2010) 38:567–9. doi: 10.1016/j.biologicals.2010.05.003 20638299

[67] Sidiropoulos PI Karvounaris SA Boumpas DT. Metabolic syndrome in rheumatic diseases: epidemiology, pathophysiology, and clinical implications. Arthritis Res Ther. (2008) 10:207. doi: 10.1186/ar2397 18492218 PMC2483433

[68] Gonzalez-Gay MA De Matias JM Gonzalez-Juanatey C Garcia-Porrua C Sanchez-Andrade A Martin J. Anti-tumor necrosis factor-alpha blockade improves insulin resistance in patients with rheumatoid arthritis. Clin Exp Rheumatol. (2006) 24:83–6. 16539824

[69] Chen HH Chen DY Lin CC Chen YM Lai KL Lin CH. Association between use of disease-modifying antirheumatic drugs and diabetes in patients with ankylosing spondylitis, rheumatoid arthritis, or psoriasis/psoriatic arthritis: a nationwide, population-based cohort study of 84,989 patients. Ther Clin Risk Manag. (2017) 13:583–92. doi: 10.2147/TCRM.S130666 PMC5422572 28496328

[70] Akdis CA. Does the epithelial barrier hypothesis explain the increase in allergy, autoimmunity and other chronic conditions? Nat Rev Immunol. (2021) 21:739–51. doi: 10.1038/s41577-021-00538-7 33846604

[71] Zhou C Zhao H Xiao XY Chen BD Guo RJ Wang Q. Metagenomic profiling of the pro-inflammatory gut microbiota in ankylosing spondylitis. J Autoimmun. (2020) 107:102360. doi: 10.1016/j.jaut.2019.102360 31806420

[72] Costello ME Ciccia F Willner D Warrington N Robinson PC Gardiner B. Brief report: intestinal dysbiosis in ankylosing spondylitis. Arthritis Rheumatol. (2015) 67:686–91. doi: 10.1002/art.38967 25417597

[73] Asquith M Sternes PR Costello ME Karstens L Diamond S Martin TM. HLA alleles associated with risk of ankylosing spondylitis and rheumatoid arthritis influence the gut microbiome. Arthritis Rheumatol. (2019) 71:1642–50. doi: 10.1002/art.v71.10 31038287

[74] Zhou H Sun L Zhang S Zhao X Gang X Wang G. Evaluating the causal role of gut microbiota in type 1 diabetes and its possible pathogenic mechanisms. Front Endocrinol (Lausanne). (2020) 11:125. doi: 10.3389/fendo.2020.00125 32265832 PMC7105744

[75] Watts T Berti I Sapone A Gerarduzzi T Not T Zielke R. Role of the intestinal tight junction modulator zonulin in the pathogenesis of type I diabetes in BB diabetic-prone rats. Proc Natl Acad Sci USA. (2005) 102:2916–21. doi: 10.1073/pnas.0500178102 PMC549484 15710870

[76] Lee AS Gibson DL Zhang Y Sham HP Vallance BA Dutz JP. Gut barrier disruption by an enteric bacterial pathogen accelerates insulitis in NOD mice. Diabetologia. (2010) 53:741–8. doi: 10.1007/s00125-009-1626-y 20012858

[77] Tajik N Frech M Schulz O Schälter F Lucas S Azizov V. Targeting zonulin and intestinal epithelial barrier function to prevent onset of arthritis. Nat Commun. (2020) 11:1995. doi: 10.1038/s41467-020-15831-7 32332732 PMC7181728

