# Supplementary material for: The harmful effect of ankylosing spondylitis on diabetes mellitus: new evidence from the Mendelian randomization analysis
Source: Front Endocrinol (Lausanne). 2024 Nov 22;15:1369466. doi: 10.3389/fendo.2024.1369466 (PMC11624504; doi:10.3389/fendo.2024.1369466)
Supplement: Supplementary file 1 [file DataSheet1.pdf]

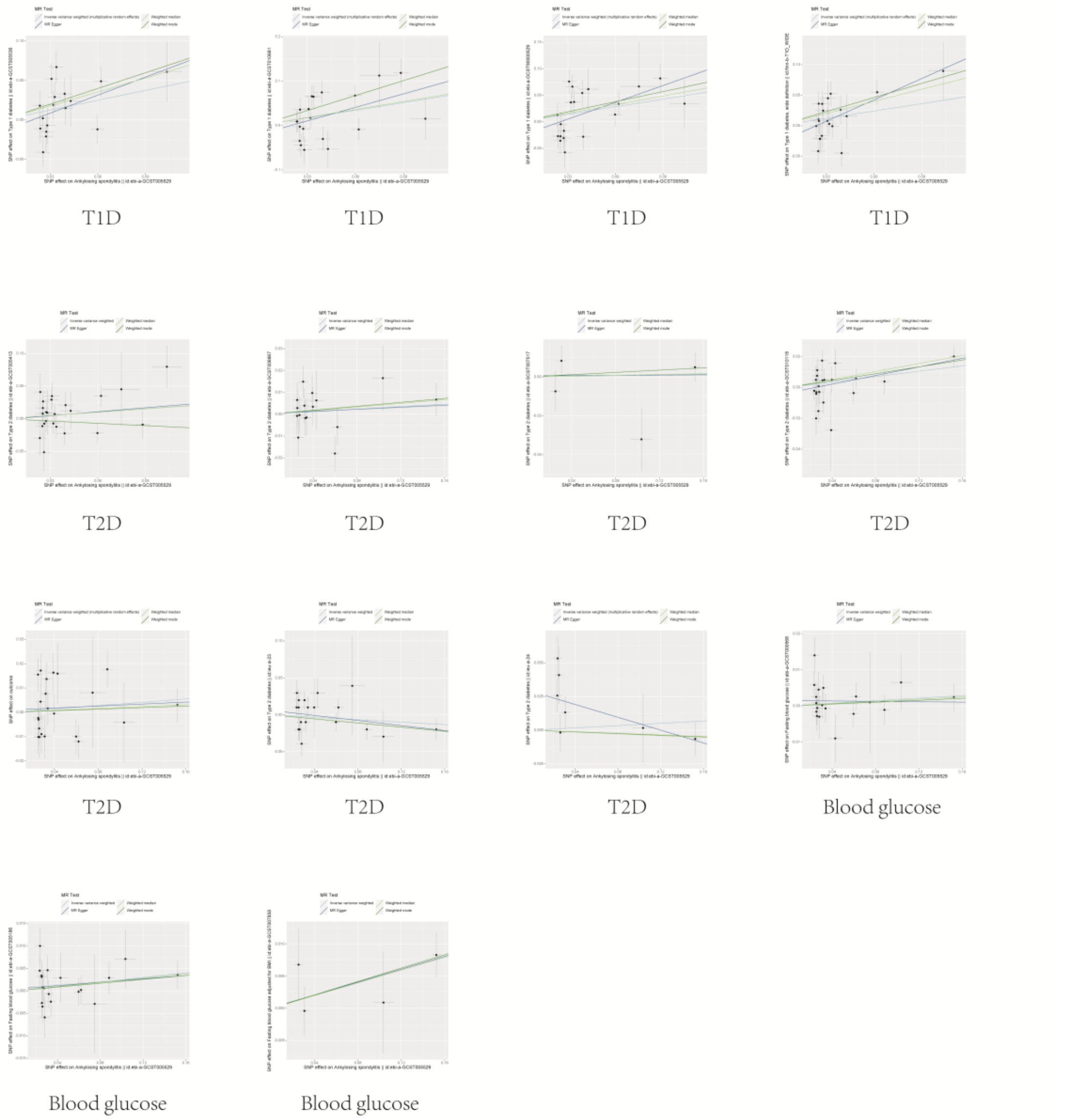

Figure S1. The Scatter plot of ankylosing spondylitis on T1D, T2D, and blood glucose. T1D: Type 1 diabetes. T2D: Type 2 diabetes.

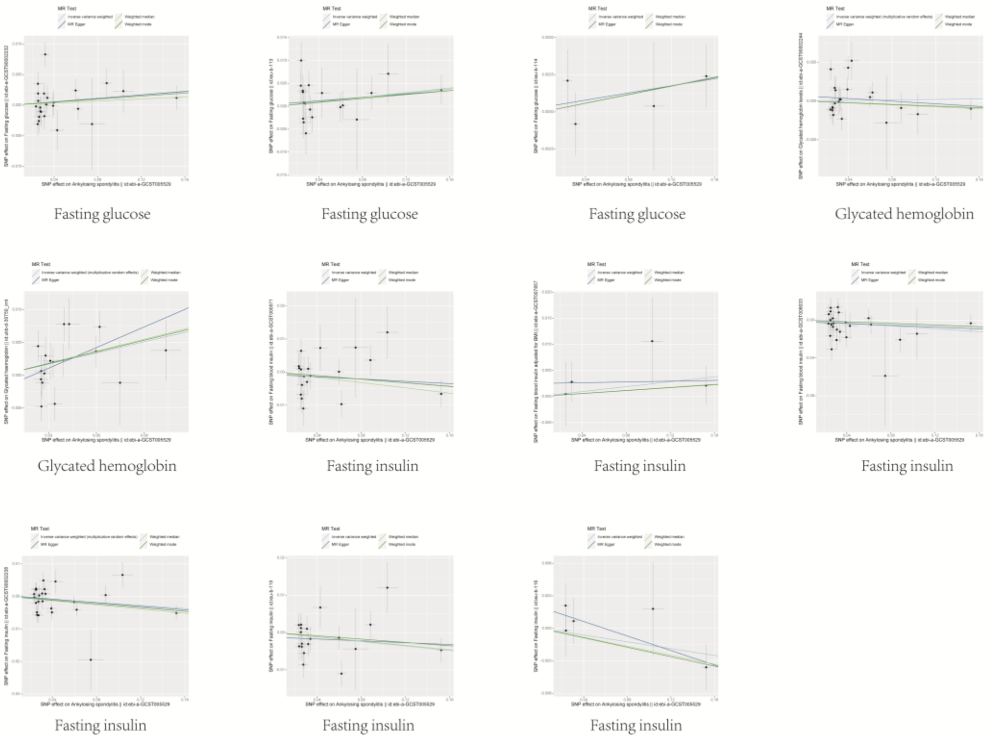

Figure S2, The scatter plot of the association between ankylosing spondylitis on fasting glucose, glycated hemoglobin, and fasting insulin.
